# Mediational Role of Executive Functions on Parental Style and Anxiety and Depression Symptoms in Adolescents From Different Vulnerability Environments: A Cross‐Sectional Study

**DOI:** 10.1155/da/1162692

**Published:** 2026-08-20

**Authors:** Anna Carballo-Márquez, Aikaterini Ampatzoglou, Juliana Rojas-Rincón, Cristina Fernández-Cardellach, Anna Garcia-Casanovas, María Fernández-Capo, María Gámiz-Sanfeliu, Maite Garolera, Bruno Porras-Garcia

**Affiliations:** ^1^ BrainXR Lab, Department of Psychology, International University of Catalonia, Sant Cugat del Vallès, Spain, uic.es; ^2^ Brain, Cognition, and Behavior Research Group, Consorci Sanitari de Terrassa-Hospital Universitari, Terrassa, Spain

**Keywords:** adolescence, anxiety, depression, executive functions, parental style, school vulnerability

## Abstract

Executive functions (EFs) and parental styles are key determinants of adolescent mental health. This study investigates the mediating role of EFs in the relationship between parenting styles (communicative, hostile, and neglectful) and anxiety and depression symptoms in a sample of 165 adolescents (50.9% boys and 49.1% girls) from 12 to 16 years old (mean age 13.29) from schools with varying socioeconomic vulnerability levels. Participants completed the Child’s Report of Parental Behavior Inventory, the Behavior Rating Inventory of Executive Function 2 and the Revised Child Anxiety and Depression Scale. The results show that adolescents in high‐vulnerability schools exhibit significantly higher anxiety and depression symptoms and perceive lower parental communication from both mothers and fathers compared to those in low‐vulnerability schools. No significant differences were observed in EFs difficulties based on school vulnerability. Regression analyses indicate that parental communication predicts lower levels of anxiety and depression symptoms, whereas paternal neglect emerges as a specific risk predictor. Mediation models reveal that EFs deficits, specifically in cognitive flexibility and working memory, serve as the primary mechanism linking parenting styles to emotional adjustment. These findings underscore the importance of parental communication as a secure environment that may foster not only cognitive development but also mentalization (or reflective functioning). We conclude that school‐based interventions should prioritize EFs training as an equity‐based tool to protect the well‐being of adolescents in high‐vulnerability settings.

## 1. Introduction

Executive functions (EFs) are higher‐order cognitive processes that enable goal‐directed behaviors, emotions, and thoughts and involve flexible and appropriate decision making and problem solving in novel contexts [1–4]. Several studies and meta‐analyses have consistently shown that better performance in EFs is related to better cognitive skills like pragmatic skills, reading comprehension, and language skills [5–7], better social skills [8, 9], better levels of physical, and mental health [1, 10], as well as with higher academic performance [11–13]. Most theoretical models identify three main separate but interrelated components within the EFs umbrella: inhibitory control, working memory, and cognitive flexibility [1, 4]. Inhibitory control refers to the ability to refrain from automatic responses and impulses, working memory is the ability to retain and manipulate information in the mind while retrieving information stored in long‐term memory, and cognitive flexibility is the ability to flexibly switch between two or more tasks or thoughts. According to Diamond [1], these would be the main EFs, and other higher cognitive processes such as reasoning, problem solving, or planning will be based on the functioning of these three basic skills.

Neuroimaging studies have shown that EFs depend primarily on the functioning of neural networks distributed in the prefrontal cortex and other related brain structures [14]. Such a prolonged maturation process and the consequent neuroplasticity of these areas make the development of EFs highly susceptible to external influences from earliest childhood until past adolescence [15, 16]. In this sense, contextual factors, environmental stimulation, as well as the quality of adult–child interactions, can have a significant impact on prefrontal cortex development and executive functioning [17–20].

Previous studies point out that parent–child interactions are one of the main contexts in which EFs development is going to take place, so parental styles and parental behaviors are going to clearly influence the child’s development and executive performance as adequate parenting provides support and external regulation in order for children to practice and internalize self‐regulatory skills [21, 22]. Parental styles are defined as the patterns of behavior that primary caregivers habitually use to interact with their children [23]. These necessarily involve parenting and nurturing patterns, such as physical care, feeding, behavioral habits, as well as responding to their moral and emotional needs, all of which critically impact their neurodevelopment and early development [24]. Many studies have related parental styles to EFs performance, such that parental styles based on positive behaviors (sensitivity or responsiveness, warmth, love, emotional support, secure attachment, synchronicity, or open communication) correlate positively with higher EFs development. Conversely, parental styles based on negative behaviors (hostility, intrusive control, inconsistent discipline, punishment, criticism, irritability, impatience, overprotection, or neglect) correlate negatively with executive performance [22, 25–27].

On the other hand, several studies have shown a significant relationship between parental styles and the mental health of children and adolescents [28, 29]. In this sense, eminently democratic or authoritative parental styles, based on communication, warmth, promotion of autonomy, and behavioral control through positive discipline, correlate negatively with the presence of internalizing symptomatology such as depressive or anxious symptoms [30, 31]. In contrast, more authoritarian parental styles, based on harsh discipline, hostility, punishment, overprotection, psychological control, and neglect, do correlate with a greater presence of anxious and depressive symptoms [32–34].

Positive parental styles (also known as democratic or authoritative) include several conditions, as mentioned above. However, democratic parental communication, marked by open dialog, emotional warmth, respect for children’s autonomy, and explaining the reasons behind rules and decisions [35], seems to be one of the most important factors in adolescent quality of life [36, 37] and in EFs development [38, 39]. Regarding negative parental styles, hostility [40, 41] and neglect [42, 43] appear to be significant contributors to EFs problems and psychopathology in children and adolescents.

On the other hand, many studies have also observed that vulnerable environments and a low socioeconomic status (SES) are variables that have a direct impact on the development of the prefrontal cortex [44, 45] and executive performance [46, 47] in both children and adolescents. In this sense, parental education predicts greater cortical thickness in the right anterior cingulate gyrus and left superior frontal gyrus, brain regions related to EFs performance, and higher SES is associated with higher white matter volume and integrity in tracts critical for EFs, such as frontoparietal connections.

While previous studies have attempted to explain the relationships between parental styles and EFs and parental styles and internalizing symptoms, separately, there is not enough evidence regarding the mediational relationship between these factors. In this sense, it is necessary to further explore the possible mediating role of EFs in the relationship between parental styles and internalizing symptoms in children and adolescents with different vulnerability backgrounds.

The present study aims to fill gaps in the existing literature by empirically examining the associations between certain parental styles (communicative, hostile, and neglectful parental styles), EFs, and internalizing symptomatology among adolescents from different vulnerability contexts. Our specific aims are as follows: (a) to evaluate whether there are differences in the assessed measures of anxiety and depression symptoms, parental styles (communicative, hostile, and neglectful) for both mothers and fathers, and EFs (inhibition, flexibility, and working memory) between adolescents from different vulnerability levels; (b) to assess whether different parental styles (communicative, hostile, and neglectful) for both mothers and fathers predict higher or lower levels of anxiety and depression symptoms in adolescents; and (c) to assess whether EFs (inhibition, flexibility, and working memory) act as a mediator in the relationship between parental styles (communicative, hostile, and neglectful) for both mothers and fathers and anxiety and depression symptoms in adolescents.

## 2. Methods

### 2.1. Participants

The study sample consisted of a total of 165 students of secondary Schools (50.9% boys and 49.1% girls), from 12 to 16 years old (mean age of 13.29 years, SD = 1.132), from six schools in the Barcelona metropolitan area (Spain).

Participants were included if they were able to understand, read, and speak Spanish and had obtained consent from their parents or legal guardians. Exclusion criteria comprised not understanding, reading, or speaking Spanish; having a severe psychiatric diagnosis (such as bipolar or psychotic disorders); presenting neurodevelopmental disorders (e.g., severe autism spectrum disorder or intellectual disability); or exhibiting physical, motor, or sensory impairments that could interfere with assessments. In the case of diagnoses, these were provided by families or the school. Among the 163 participants with valid RCADS total internalizing T‐scores, 32 adolescents (19.6%) scored at or above 65. Of these, 17 participants (10.4%) were in the elevated or borderline range (T‐scores = 65–69), and 15 participants (9.2%) were in the clinically elevated range (T‐scores ≥ 70).

They were assigned to a high‐ or low‐vulnerability group based on the complexity of the school they attend. The Catalan Department of Education classifies schools into four vulnerability levels (low, medium, high, and maximum) based on a socioeconomic and administrative numerical index [48]. Schools with standard or medium vulnerability (low vulnerability, *n* = 119) operate in stable environments with favorable or balanced family backgrounds and standard resources. In contrast, high‐ and maximum vulnerability schools (high‐vulnerability, *n* = 46) concentrate large numbers of vulnerable students, newly arrived immigrants, and families facing unemployment or low education levels, which grants them extra funding, smaller class sizes, and specialized support staff to guarantee educational equity.

The present study used baseline data collected as part of the broader E‐Emotio project. No separate a priori power analysis was conducted specifically for the cross‐sectional regression and mediation analyses reported here; consequently, the mediation findings were considered exploratory and interpreted cautiously. Of the 285 students initially approached, 165 met the project’s eligibility criteria and provided both adolescent assent and parent or legal guardian consent. The final baseline sample, therefore, comprised 165 adolescents (see Section 2.4 for sample analytic results).

### 2.2. Instruments

To assess parental styles, the abbreviated Spanish version of the Child’s Report of Parental Behavior Inventory (CRPBI) [49] validated in the Spanish population by Samper et al. [50] and abbreviated by Valiente et al. [51] was administered. The CRPBI is a self‐report questionnaire designed to assess parental practices perceived by children and adolescents. The abbreviated form (CRPBI‐A) consists of 29 items in a three‐point frequency response format (“Never or almost never” = 1, “Only sometimes” = 2, and “Many times” = 3) distributed in six scales that assess six styles of parental practices: communicative, hostile/rejection, controlling, permissive, overprotective, and neglectful. In the present study, only the communicative scale will be used as a positive parental style and the hostile and neglectful scales as negative parental styles since these are the characteristics that have the higher empirical support in previous studies. The internal consistency (Cronbach’s alpha coefficient) of each scale was: communicative (α = 0.86/0.82), hostile (α = 0.74/0.72), and neglectful (α = 0.57/0.50), which can be considered a serious psychometric limitation. In this sense, results involving the neglectful scale should be interpreted with considerable caution.

Regarding the assessment of EFs, the Behavior Rating Inventory of Executive Function 2 (BRIEF‐2), in its Spanish adaptation [52], was administered to the participants. The self‐reported BRIEF‐2 is a 55‐item questionnaire that assesses perceived everyday EFs difficulties with ecological validity for children and adolescents aged 5–18 years. The items are answered in frequency format on how problematic the behaviors have been during the last 6 months (“Never,” “Sometimes,” and “Frequently”), so higher scores indicate more EFs difficulties. Its correction provides seven different subscales: inhibition, working memory, flexibility, emotional control, self‐monitoring, task completion, and planning. The Cronbach’s α coefficient as an indication for internal consistency is α = 0.93, and the test–retest correlations’ mean is 0.74. In the present study, only inhibition, working memory, and flexibility subscales will be used as they are supposed to be the principal EFs [1, 4].

For the assessment of anxiety and depression symptomatology, the Revised Child Anxiety and Depression Scale (RCADS) [53] was administered in its Spanish adaptation [54]. The RCADS is a 47‐item self‐report questionnaire designed to assess the symptoms of anxiety and depressive disorders. The child or adolescent must answer the items according to a 0–3‐point frequency scale (0 = “never,” 1 = “sometimes,” 2 = “often,” and 3 = “always”), and standardized scores are obtained for the following subscales: separation anxiety disorder, social phobia, generalized anxiety disorder, panic disorder, obsessive–compulsive disorder, and major depressive disorder. The internal consistency for the Spanish version of the scale was satisfactory (Cronbach α = 0.89) [55]. In the present study, only the T‐scores for general anxiety and depression symptoms (the sum of all six subscales) were used.

We also calculated the internal consistency reliability coefficients (Cronbach’s alpha) for all the main study variables using the present sample, and they were very similar to the original ones: CRPBI‐A communicative (α = 0.85/0.85), hostile (α = 0.68/0.72), and neglectful (α = 0.54/0.52) for father and mother, respectively; BRIEF‐2 inhibition (α = 0.73), working memory (α = 0.83), and flexibility (α = 0.80) subscales; RCADS total internalizing scale (α = 0.94).

### 2.3. Procedure

This exploratory study forms part of the larger E‐emotio project, which aims to prevent anxiety and depressive disorders and to enhance associated emotional and cognitive processes through a virtual reality–based cognitive training program. The present study focuses only on a subset of baseline measures collected within this ongoing research project. The study was reviewed and approved by the Research Ethics Committee of the Universitat Internacional de Catalunya (Ref. PSI‐2023‐04), and informed consent was obtained from all participating families and students.

Participant recruitment took place between January 2024 and January 2026. The research team first contacted the dean or administrative teams of the participating schools to obtain institutional approval. Once initial approval was granted by the institution, the tutors of each participating course and the school psychologist were informed. Subsequently, families were provided with detailed information through an online session where any questions or concerns could be addressed, while students were informed in face‐to‐face sessions conducted at their schools.

Following consent, members of the research team jointly reviewed with the school team, including tutors and the school psychologist, to ensure that all participants met the inclusion requirements. Then, research assistants, including master’s and PhD‐level psychologists, visited the schools to administer the baseline assessment session during non‐lective hours, which lasted between 1.5 h per student and included the administration of several paper‐based measures related to mental health and cognitive functioning.

### 2.4. Data Analysis

Statistical analyses were conducted using IBM SPSS Statistics for Windows and the PROCESS macro for mediation analyses. All statistical tests were two‐tailed, with the significance level set at *p* < 0.05. First, descriptive statistics were calculated for all study variables. Bivariate correlations were then examined among anxiety and depression symptoms, maternal and paternal parenting‐style dimensions, and EF dimensions. Pearson’s correlation coefficients were calculated when parametric assumptions were adequately met; otherwise, Spearman’s rank‐order correlations were used.

To address the first specific aim, participants were classified according to the complexity level of the school they attended and divided into low‐ and high‐vulnerability groups. Differences between the low‐ and high‐vulnerability school groups in anxiety and depressive symptoms, maternal and paternal parenting styles, and EF dimensions were examined using Mann–Whitney *U* tests because most study variables did not satisfy the normality assumption.

To address the second specific aim, hierarchical multiple linear regression analyses were conducted to examine whether communicative, hostile, and neglectful parental styles were associated with adolescents’ anxiety and depression symptoms after accounting for available sociodemographic characteristics (age and sex) and the school complexity level. Separate models were estimated for maternal and paternal parenting styles. In each model, age, sex, and school complexity level were entered in the first block, followed by the three corresponding parenting‐style dimensions, communication, hostility, and neglect, in the second block. The assumptions of linear regression were adequately met for both models. For the maternal model, no problematic multicollinearity was observed, with tolerance values ranging from 0.566 to 0.928 and VIF values ranging from 1.08 to 1.77. The independence of errors was supported by a Durbin–Watson statistic of 2.00, and no highly influential observations were identified, with a maximum Cook’s distance of 0.252. Inspection of the residual plots supported linearity and homoscedasticity, and the residuals were approximately normally distributed. For the paternal model, collinearity diagnostics likewise indicated no problematic multicollinearity, with tolerance values ranging from 0.47 to 0.93 and VIF values ranging from 1.07 to 2.11. The Durbin–Watson statistic was 2.09, and Cook’s distance values remained well below 1. Inspection of the residual plots supported linearity and homoscedasticity, and the residuals were approximately normally distributed.

**Figure 1:**
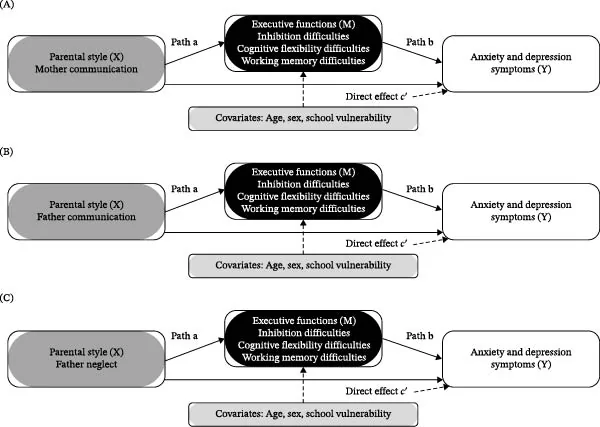
Study mediational model of executive functions on the relationship between parental style and internalizing symptoms. (A) Maternal communication; (B) paternal communication; (C) paternal neglect. *Note*: Path a represents the association between X and each mediator; Path b represents the association between each mediator and Y while controlling for X, the other mediators, and the covariates; *c*′ represents the direct effect of X on Y after accounting for the mediators.

To address the third specific aim, mediation analyses were conducted to examine whether EF dimensions mediated the relationships between the parenting dimensions that showed significant associations with anxiety and depressive symptoms in the preceding adjusted regression analyses. Parallel multiple‐mediator models were estimated using PROCESS Model 4. In each model, one parenting‐style dimension was entered as the independent variable (X), anxiety and depressive symptoms as the dependent variable (Y), and inhibition, cognitive flexibility, and working memory as three simultaneous parallel mediators (M1, M2, and M3, respectively). Age, sex, and school complexity level were included as covariates (Figure 1). Indirect effects were estimated using percentile bootstrap confidence intervals based on 5000 bootstrap samples. The *a* path from the parenting variable to each executive‐function mediator, the *b* paths from each mediator to anxiety and depressive symptoms, the total effect (*c*), the direct effect (*c′*), and the total and specific indirect effects were reported. An indirect effect was considered statistically significant when its 95% bootstrap confidence interval did not include zero.

Missing data were minimal, and analytical sample sizes varied according to the variables required for each analysis. Bivariate correlations (*N*s ranging from 161 to 165) were estimated using pairwise deletion, such that each correlation was based on all participants with valid data for the corresponding pair of variables. Hierarchical regression and indirect‐effect analyses used complete case analysis. Because the indirect‐effect models simultaneously required valid scores for all three BRIEF‐2 dimensions, the parenting variable, RCADS internalizing symptoms, and all covariates, their analytic samples were slightly smaller. The maternal communication model included 160 participants, whereas the paternal communication and paternal neglect models each included 159 participants. No missing values were imputed.

## 3. Results

Descriptive statistics, including means and standard deviations, 95% confidence intervals, medians, and interquartile ranges, for anxiety and depressive symptoms, maternal and paternal parenting styles, and self‐reported EF difficulties are presented in Table 1. Mann–Whitney *U* tests identified significant differences by school complexity level in anxiety and depressive symptoms and perceived parental communication. Adolescents attending high‐vulnerability schools reported higher anxiety and depressive symptoms than those attending low‐complexity schools. In contrast, adolescents attending low‐vulnerability schools reported higher maternal and paternal communication than those attending high‐vulnerability schools. No significant group differences were observed for inhibition, cognitive flexibility, working memory, or hostile and neglectful maternal or paternal parenting styles (see Table 1).

**Table 1:** Descriptive statistics and Mann–Whitney *U* comparisons.

Measure	Low‐vulnerability schools			High‐vulnerability schools			*U*	*z*	*p*
	*M* (SD)	95% CI	Mdn (IQR)	*M* (SD)	95% CI	Mdn (IQR)			
Anxiety and depressive symptoms	53.30 (10.51)	[51.35, 55.25]	52.00 (16)	57.37 (11.81)	[53.86, 60.88]	57.00 (15)	3265.00	2.12	**0.034**
Inhibition difficulties	12.41 (2.87)	[11.88, 12.94]	12.00 (4)	11.59 (3.95)	[10.41, 12.76]	12.00 (5)	2541.00	−0.64	0.524
Cognitive flexibility difficulties	14.62 (3.45)	[13.98, 15.26]	14.00 (5)	14.89 (3.09)	[13.97, 15.81]	16.00 (4)	3002.50	1.15	0.248
Working memory difficulties	14.64 (3.96)	[13.91, 15.38]	14.00 (6)	14.89 (2.92)	[14.02, 15.76]	15.00 (5)	2951.50	0.95	0.335
Mother communication	17.67 (3.52)	[17.01, 18.32]	19.00 (4)	16.20 (4.34)	[14.91, 17.48]	17.00 (7)	2060.00	−2.48	**0.013**
Mother hostility	7.64 (2.31)	[7.21, 8.07]	7.00 (3)	7.91 (2.51)	[7.17, 8.66]	8.00 (4)	3019.00	1.04	0.300
Mother neglect	5.44 (1.57)	[5.15, 5.73]	5.00 (2)	5.74 (1.89)	[5.18, 6.30]	5.00 (2)	3095.50	1.34	0.179
Father communication	16.33 (4.43)	[15.51, 17.16]	18.00 (6)	13.39 (6.70)	[11.40, 15.38]	14.50 (8)	1968.50	−2.74	**0.006**
Father hostility	7.00 (2.36)	[6.56, 7.44]	7.00 (2)	6.13 (3.35)	[5.14, 7.13]	6.50 (3)	2399.00	−1.17	0.242
Father neglect	5.66 (1.90)	[5.31, 6.01]	5.00 (1)	5.22 (2.71)	[4.41, 6.02]	5.00 (3)	2719.50	0.02	0.984

*Note*: Anxiety and depressive symptoms were assessed using the RCADS total internalizing scale. Executive‐function difficulties were assessed using the self‐report BRIEF‐2 inhibition, cognitive flexibility, and working memory subscales. Parenting styles were assessed using the CRPBI‐A. Higher executive‐function scores indicate greater reported difficulties. Two‐sided *p* values are reported. Statistically significant *p* values (*p* < .05) are shown in bold.

Abbreviations: CI, confidence interval; IQR, interquartile range; M, mean; Mdn, median; SD, standard deviation; U, Mann–Whitney *U* statistic.

Spearman’s rank‐order correlations among the study variables are presented in Table 2. Anxiety and depressive symptoms were significantly associated with all three EF dimensions, with the strongest association observed for cognitive flexibility difficulties, followed by working memory and inhibition difficulties. Regarding parenting styles, higher anxiety and depressive symptoms were associated with lower perceived maternal and paternal communication and with higher levels of maternal and paternal hostility and neglect. Strong positive correlations were also observed between corresponding maternal and paternal parenting dimensions, particularly communication and neglect, and between paternal hostility and paternal neglect.

**Table 2:** Spearman correlations among anxiety and depression symptoms, executive‐function dimensions, and parenting‐style dimensions.

Variable	1	2	3	4	5	6	7	8	9	10
1. Anxiety and depression symptoms	—	—	—	—	—	—	—	—	—	—
2. Inhibition	0.352 ^∗∗∗^	—	—	—	—	—	—	—	—	—
3. Cognitive flexibility	0.583 ^∗∗∗^	0.510 ^∗∗∗^	—	—	—	—	—	—	—	—
4. Working memory	0.482 ^∗∗∗^	0.516 ^∗∗∗^	0.659 ^∗∗∗^	—	—	—	—	—	—	—
5. Paternal communication	−0.342 ^∗∗∗^	−0.230 ^∗∗^	−0.285 ^∗∗∗^	−0.405 ^∗∗∗^	—	—	—	—	—	—
6. Paternal hostility	0.194 ^∗^	0.233 ^∗∗^	0.180 ^∗^	0.182 ^∗^	−0.165 ^∗^	—	—	—	—	—
7. Paternal neglect	0.297 ^∗∗∗^	0.208 ^∗∗^	0.266 ^∗∗^	0.209 ^∗∗^	−0.302 ^∗∗∗^	0.542 ^∗∗∗^	—	—	—	—
8. Maternal communication	−0.315 ^∗∗∗^	−0.257 ^∗∗^	−0.285 ^∗∗∗^	−0.363 ^∗∗∗^	0.682 ^∗∗∗^	−0.255 ^∗∗^	−0.338 ^∗∗∗^	—	—	—
9. Maternal hostility	0.284 ^∗∗∗^	0.368 ^∗∗∗^	0.318 ^∗∗∗^	0.346 ^∗∗∗^	−0.326 ^∗∗∗^	0.392 ^∗∗∗^	0.281 ^∗∗∗^	−0.430 ^∗∗∗^	—	—
10. Maternal neglect	0.255 ^∗∗^	0.177 ^∗^	0.163 ^∗^	0.232 ^∗∗^	−0.351 ^∗∗∗^	0.283 ^∗∗∗^	0.598 ^∗∗∗^	−0.358 ^∗∗∗^	0.396 ^∗∗∗^	—

*Note*: Higher scores on the EF dimensions indicate greater executive‐function difficulties. Analytic sample *N* = 161–165.

^∗^ *p*  < 0.05.

^∗∗^ *p*  < 0.01.

^∗∗∗^ *p*  < 0.001.

### 3.1. Hierarchical Regression Analyses

#### 3.1.1. Maternal Parenting Styles

In the first step, age, sex, and school complexity level jointly explained 5.5% of the variance in anxiety and depression symptoms, *R* ^2^ = 0.055, adjusted *R* ^2^ = 0.037, *F* (3, 159) = 3.08, *p* = 0.029. The addition of maternal communication, hostility, and neglect in the second step explained a significant additional 7.8% of the variance, Δ*R* ^2^ = 0.078, Δ*F*(3, 156) = 4.71, *p* = 0.004. The final model was statistically significant, *F* (6, 156) = 4.01, *p* = 0.001, accounting for 13.3% of the variance in anxiety and depression symptoms (*R* ^2^ = 0.133, adjusted *R* ^2^ = 0.100).

In the fully adjusted model, higher maternal communication was significantly associated with lower anxiety and depression symptoms, *B* = −0.46, SE = 0.23, *β* = −0.16, *p* = 0.047, 95% CI [−0.91, −0.01]. Maternal hostility and maternal neglect were not significant independent predictors (*p* > 0.05) after accounting for the covariates and the other maternal parenting dimensions. The school complexity level and sex, which were significant in the first step, were no longer statistically significant in the final model.

#### 3.1.2. Paternal Parenting Styles

In the first step, age, sex, and school complexity level explained 5.5% of the variance in anxiety and depression symptoms, *R* ^2^ = 0.055, adjusted *R* ^2^ = 0.037, *F* (3, 158) = 3.06, *p* = 0.030. After paternal communication, hostility, and neglect were entered in the second step, the full model explained 16.9% of the variance, *R* ^2^ = 0.169, adjusted *R* ^2^ = 0.136, *F* (6, 155) = 5.24, *p*  < 0.001. The addition of paternal parenting‐style dimensions explained a significant additional 11.4% of the variance in anxiety and depression symptoms, Δ*R* ^2^ = 0.114, Δ*F*(3, 155) = 7.07, *p*  < 0.001.

In the final adjusted model, higher paternal communication was significantly associated with lower anxiety and depression symptoms, *B* = −0.55, SE = 0.16, *β* = −0.27, *p* = 0.001, 95% CI [−0.88, −0.23]. Conversely, higher paternal neglect was significantly associated with higher anxiety and depression symptoms, *B* = 1.42, SE = 0.54, *β* = 0.28, *p* = 0.009, 95% CI [0.36, 2.49]. Paternal hostility was not significantly associated with anxiety and depression symptoms after accounting for the covariates and the other paternal parenting‐style dimensions. None of the covariates was significantly associated with anxiety and depression symptoms in the final model.

### 3.2. Parallel Mediation Analyses

Based on the preceding statistically significant adjusted regression analyses, three parallel mediation models were conducted to examine whether EFs difficulties mediated the associations of maternal communication, paternal communication, and paternal neglect with adolescents’ anxiety and depressive symptoms. In each model, inhibition, cognitive flexibility, and working memory were entered simultaneously as parallel mediators, while age, sex, and school complexity level were included as covariates. The complete results are presented in Table 3.

**Table 3:** Adjusted parallel mediation models of executive‐function difficulties in the associations between parenting styles and anxiety and depression symptoms.

Model	Path	Predictor → Outcome	*B*	SE	95% CI	*p*
Panel A: Path coefficients, total effects, and direct effects						
Mother communication	*a*_1_	Mother communication → Inhibition	−0.21	0.07	[−0.35, −0.08]	<0.01
	*a*_2_	Mother communication → Cognitive flexibility	−0.18	0.07	[−0.32, −0.05]	0.01
	*a*_3_	Mother communication → Working memory	−0.29	0.07	[−0.44, −0.14]	<0.001
	*b*_1_	Inhibition → Internalizing symptoms	−0.04	0.27	[−0.57, 0.50]	0.89
	*b*_2_	Cognitive flexibility → Internalizing symptoms	1.42	0.29	[0.84, 1.99]	<0.001
	*b*_3_	Working memory → Internalizing symptoms	0.55	0.27	[0.02, 1.08]	0.04
	*c*	Mother communication → Internalizing symptoms	−0.58	0.23	[−1.03, −0.14]	0.01
	*c′*	Mother communication → Internalizing symptoms	−0.18	0.20	[−0.58, 0.23]	0.39
Father communication	*a*_1_	Father communication → Inhibition	−0.07	0.05	[−0.17, 0.03]	0.17
	*a*_2_	Father communication → Cognitive flexibility	−0.09	0.05	[−0.19, 0.01]	0.07
	*a*_3_	Father communication → Working memory	−0.20	0.05	[−0.31, −0.09]	<0.001
	*b*_1_	Inhibition → Internalizing symptoms	−0.03	0.26	[−0.55, 0.49]	0.90
	*b*_2_	Cognitive flexibility → Internalizing symptoms	1.42	0.29	[0.85, 1.98]	<0.001
	*b*_3_	Working memory → Internalizing symptoms	0.63	0.27	[0.10, 1.16]	0.02
	*c*	Father communication → Internalizing symptoms	−0.40	0.17	[−0.73, −0.06]	0.02
	*c′*	Father communication → Internalizing symptoms	−0.14	0.15	[−0.43, 0.15]	0.34
Father neglect	*a*_1_	Father neglect → Inhibition	0.30	0.12	[0.07, 0.53]	0.01
	*a*_2_	Father neglect → Cognitive flexibility	0.28	0.12	[0.04, 0.51]	0.02
	*a*_3_	Father neglect → Working memory	0.22	0.13	[−0.04, 0.48]	0.10
	*b*_1_	Inhibition → Internalizing symptoms	−0.10	0.26	[−0.62, 0.42]	0.70
	*b*_2_	Cognitive flexibility → Internalizing symptoms	1.35	0.29	[0.79, 1.92]	<0.001
	*b*_3_	Working memory → Internalizing symptoms	0.70	0.26	[0.19, 1.21]	0.01
	*c*	Father neglect → Internalizing symptoms	1.15	0.39	[0.37, 1.92]	<0.01
	*c′*	Father neglect → Internalizing symptoms	0.64	0.33	[−0.01, 1.30]	0.05

Model		Indirect pathway	*ab*	*BootSE*	95% bootstrap CI	*abcs*
Panel B: Total and specific indirect effects						
Mother communication		Total indirect effect	−0.41	0.16	[−0.74, −0.12]	−0.14
		Via inhibition	0.01	0.07	[−0.14, 0.14]	0.00
		Via cognitive flexibility	−0.26	0.11	[−0.48, −0.06]	−0.09
		Via working memory	−0.16	0.09	[−0.36, 0.00]	−0.06
Father communication		Total indirect effect	−0.26	0.10	[−0.47, −0.09]	−0.12
		Via inhibition	0.00	0.03	[−0.07, 0.05]	0.00
		Via cognitive flexibility	−0.13	0.07	[−0.29, −0.01]	−0.06
		Via working memory	−0.13	0.06	[−0.25, −0.02]	−0.06
Father neglect		Total indirect effect	0.50	0.26	[0.04, 1.08]	0.10
		Via inhibition	−0.03	0.10	[−0.24, 0.17]	−0.01
		Via cognitive flexibility	0.38	0.18	[0.07, 0.79]	0.07
		Via working memory	0.15	0.11	[−0.03, 0.41]	0.03

*Note*: All models were estimated using PROCESS Model 4 and adjusted for school complexity level, age, and sex. Percentile bootstrap confidence intervals were based on 5000 bootstrap samples. Indirect effects were considered statistically significant when their 95% bootstrap confidence intervals did not include zero. Analytic sample *N* = 159–160 (based on complete‐case analysis for mediation models).

Abbreviations: ab, unstandardized indirect effect; abcs, completely standardized indirect effect; B, unstandardized regression coefficient; BootSE, bootstrap standard error; *c*′, direct effect; *c*, total effect; CI, confidence interval; SE, standard error.

For maternal communication, higher perceived communication was significantly associated with fewer difficulties in inhibition, cognitive flexibility, and working memory. In the simultaneous mediator model, cognitive flexibility and working memory difficulties were independently associated with higher anxiety and depressive symptoms, whereas inhibition was not. The total indirect effect was significant. A significant specific indirect effect was observed through cognitive flexibility but not through inhibition. The indirect effect through working memory had an upper confidence‐limit bound of 0.00 and was therefore not interpreted as significant. The total association between maternal communication and anxiety and depressive symptoms was significant, but the direct association was no longer significant after the EF mediators were included.

For paternal communication, higher perceived communication was significantly associated with fewer working memory difficulties. Its associations with inhibition and cognitive flexibility difficulties were not statistically significant. Cognitive flexibility and working memory difficulties were independently associated with higher anxiety and depressive symptoms, whereas inhibition was not. Significant specific indirect effects were observed through both cognitive flexibility and working memory but not through inhibition. The total indirect effect was also significant. The total association between paternal communication and anxiety and depressive symptoms was significant, whereas the direct association was no longer significant after accounting for the EF mediators.

For paternal neglect, greater perceived neglect was significantly associated with greater inhibition and cognitive flexibility difficulties but not with working memory difficulties. Cognitive flexibility and working memory difficulties were independently associated with higher anxiety and depressive symptoms, whereas inhibition was not. The total indirect effect was significant. However, only the specific indirect effect through cognitive flexibility was significant; the indirect effects through inhibition and working memory were not significant. The total association between paternal neglect and anxiety and depressive symptoms was significant, whereas the direct association was not statistically significant after the EF mediators were included.

## 4. Discussion and Conclusions

The aim of the present study was to examine the association between different parental styles, with possible deficits in EFs, anxiety and depression symptomatology, and social vulnerability in adolescents. The results obtained showed that adolescents attending low‐vulnerability schools exhibited fewer symptoms of anxiety and depression than those attending high‐vulnerability schools. This finding is consistent with previous studies that used SES as a measure of social vulnerability related to a significant effect on children and adolescents’ mental health [56, 57]. The Lemstra et al. (2008) systematic review validates our findings by demonstrating a clear inverse association between SES and mental health symptoms in youth aged 10–15. By pooling data from over 34,000 participants, the study found that adolescents from low SES backgrounds are ~2.49 times more likely to suffer from a depressed mood or anxiety compared to those with higher SES. This evidence directly supports our observation that students in high‐vulnerability schools—often characterized by lower socioeconomic indicators—present significantly higher signs of anxiety and depression than those in low‐vulnerability settings.

Adolescents from high‐vulnerability schools also reported significantly lower levels of perceived communicative parenting styles from both their mothers and fathers than adolescents from low‐vulnerability schools. This result is consistent with previous research observing a lower presence of the father communicative parental style in low SES groups [58]. The research suggests that in low SES or high‐vulnerability contexts, financial and social stressors can become limiting factors that hinder a parent’s ability to maintain this communicative and supportive environment [59]. Some authors have documented that parents in low SES families are harsher and more punitive [60] because of different mediating variables like parental knowledge and expectations, parental distress or mental health, access to resources, and cultural norms and values [61], which could also be present in our study.

On the other hand, no differences were observed in self‐reported EFs problems between adolescents from low‐ and high‐vulnerability schools, which contrasts with the extensive research linking SES and other social vulnerability variables to EFs performance [46, 62]. However, in our study, we assessed EFs through a self‐report questionnaire rather than cognitive tests that measure executive performance objectively. This may explain why we did not observe any differences between groups, as some studies have pointed out [63].

Correlation analyses showed that anxiety and depressive symptoms were significantly associated with all three EFs dimensions, with the strongest association observed for cognitive flexibility difficulties, followed by working memory and inhibition difficulties. This correlation is strongly supported by previous research observing robust links between higher anxiety and depression symptom severity and lower reported EFs abilities in adolescents [64–67]. This relationship is supported by a possible impairment of prefrontal cortex functioning in mood disorders [68] and an impaired capacity for self‐care in people with anxiety and depression disorders [69].

Regarding parental styles’ correlations of both mother and father were associated with higher or lower anxiety and depression symptomatology in adolescents. In line with previous studies, a parental style that is positive and communication‐based can prevent the onset of anxiety and depression symptoms in children [70] and adolescents [71]. When these associations were analyzed after accounting for available sociodemographic characteristics (age and sex) and school vulnerability level, only mother and father communication parental style and father neglect parental style were still significant independent predictors of anxiety and depression symptoms in our sample. Communicative parental styles may be a protective factor because responsive parents can identify their children’s issues and provide help and emotional support, thereby reducing the likelihood of internalizing symptoms [28]. Similarly, dialogic and communicative parents may model adaptive emotional regulation strategies based on expressiveness and emotion management rather than suppression or avoidance for their children [72], which has also been found to correlate positively with a lower presence of anxiety and depression symptoms in adolescents [73]. Conversely, previous studies have found that a negative parental style is associated with the onset of mental health problems in children and adolescents [74]. The impact of coercive and over‐reactive parenting on internalizing and externalizing problems in children and adolescents may be due to its effect on self‐esteem [75], self‐control [76], and emotion regulation [77]. A neglectful parental style is also associated with mental health issues in children and adolescents [29] as the unresponsive care can leave them with models of themselves as unworthy of love and others as unavailable or rejecting [78]. Compared to children who have experienced physical abuse, those who have been neglected exhibit more severe cognitive and academic deficits, social withdrawal, limited peer interactions, and anxiety and depression problems [79].

Mediation model findings obtained in our study can enhance our understanding of how EFs deficits mediate the predictive effect of positive and negative parental practices on adolescents’ anxiety and depression symptoms. Across the three mediation models, cognitive flexibility emerged as the most consistent mediator of these associations. Specifically, greater maternal communication and lower paternal neglect were associated with fewer cognitive flexibility difficulties, in line with previous research [80], which in turn were related to lower levels of anxiety and depressive symptoms. In contrast, inhibition did not significantly mediate any of the examined associations. Although maternal communication and paternal neglect were related to inhibition difficulties, inhibition did not independently predict anxiety and depressive symptoms once cognitive flexibility and working memory were considered simultaneously. This pattern suggests that inhibitory control may share variance with other EFs domains [4] but does not uniquely explain the association between parenting and anxiety and depression symptoms in this sample. Although inhibitory control has been associated with anxiety and depression symptoms in previous research [81–83], its unique contribution may be attenuated when other executive processes, such as cognitive flexibility and working memory, are considered simultaneously.

Working memory showed a more nuanced pattern. Difficulties in working memory were consistently associated with greater anxiety and depressive symptoms [84]; however, its mediating role was only supported in the association with paternal communication. Together, these findings suggest that while working memory may contribute to adolescents’ emotional functioning [85], cognitive flexibility appears to play a more central role in the pathway linking parenting experiences to anxiety and depression symptoms [86].

The prominent role of cognitive flexibility may reflect its importance in adapting to changing environmental demands, shifting between perspectives, and generating alternative responses to stressful situations [87]. Adolescents with greater cognitive flexibility may therefore be better equipped to regulate emotional responses and cope with interpersonal challenges, reducing their vulnerability to anxiety and depressive symptoms [88, 89]. Conversely, supportive parenting practices, particularly effective communication, may foster the development of cognitive flexibility by providing emotionally secure environments in which adolescents can learn adaptive problem‐solving and emotion‐regulation strategies [18, 90]. In contrast, neglectful parenting may hinder the development of these cognitive processes, increasing vulnerability to emotional difficulties [42, 91]. Beyond the direct impact on EFs, the results of this study regarding communicative parenting could be enriched through a mentalization‐based framework [92]. In this sense, Sharp and Fonagy (2008) suggest that communicative parenting is not only essential for EF development but also fundamental for fostering reflective functioning. This capacity of the adolescent to understand their own and others’ behavior in terms of underlying mental states offers a complementary explanation: parental reflective communication may facilitate the adolescent’s ability to understand and regulate internal emotional states, a process central to both the mentalization theory and the adaptive use of cognitive flexibility.

Finally, the finding that the direct associations between parenting variables and anxiety and depressive symptoms were no longer statistically significant after accounting for EFs difficulties suggests that these cognitive processes may constitute an important pathway through which parenting is associated with adolescents’ emotional adjustment. In a recent study, researchers examined whether EFs mediate the relationship between parental styles and externalizing behavior problems in children from 5 to 9 years old [93]. The results obtained showed statistically significant indirect effects between parental styles with high negativity (authoritarian and neglectful styles) on externalizing problems through deficits in EFs but not so for positive parental styles (warm and democratic styles). A direct and indirect effect of a hostile or aggressive parental style on the appearance of externalizing symptomatology through EFs deficits was also observed, concluding that EFs difficulties could be contributing to the social impairments observed in children whose parents show high parental negativity.

The present study has observed that parental styles affect adolescents’ anxiety and depression symptoms through their impact on EFs. While EFs development is related to parental styles [27], both in children [25, 94] and adolescents [26], as also reported in our study, EFs depend also on many other factors that can enhance or diminish their development as computerized training, mindfulness, some adapted curricula or physical exercise noncomputerized games, aerobics, martial arts, yoga, mindfulness, and school curricula [95]. Our results also emphasize the importance of enhancing EFs as a protective factor against the development of anxiety and depression symptoms in adolescence, given their relation to emotional regulation [96, 97] and/or as a significant protective factor against anxiety and depression symptoms [98, 99]. To this end, interventions, policies, and educational practices should focus on training programs to enhance children’s and adolescents’ EFs through childhood and adolescence [100, 101].

Despite the significant findings of this study, it is crucial to consider several limitations that could influence its interpretation and generalization. First, the study did not collect specific socioeconomic information about our participants that would allow us to make a better distinction between SES groups like parental education, income, occupation, or neighborhood [102], nor were we able to obtain comparable group measurements to enable us to perform a proper analysis. Second, EFs were assessed indirectly through adolescents’ self‐report using the BRIEF‐2 rather than standardized neuropsychological tests [22]. This approach was chosen because an ecologically valid, contextually grounded assessment of EFs was considered more appropriate for capturing the impact of EFs on adolescents’ everyday functioning [103], whereas traditional neuropsychological measures primarily assess isolated cognitive processes under controlled conditions. Nevertheless, relying on self‐report measures may have introduced common‐method variance and shared response biases, potentially inflating the observed associations. Future studies should therefore replicate and extend these findings using standardized neuropsychological assessments of EFs. In addition, the assessment focused on EFs deficits rather than the executive competencies. As the absence of deficits does not necessarily imply the presence of strengths or well‐developed skills [41], future research should examine whether positive EFs are differentially associated with parenting styles and symptoms of anxiety and depression. Finally, the exclusive use of adolescents’ self‐reports did not allow for the inclusion of a broader family perspective, including parent‐reported measures of EFs or parenting practices. Although incorporating multiple informants would provide a more comprehensive assessment, the use of adolescent self‐report was considered appropriate to minimize potential biases arising from parents’ underestimation or overestimation of their children’s EFs [104, 105]. In contrast, assessing parental styles through a questionnaire completed by the adolescents themselves increases reliability, as other studies in which parents assessed their own parental style produced inconsistent results [26]. However, the CRPBI neglectful scale showed low internal consistency, which can be considered a serious psychometric limitation.

Thirdly, the cross‐sectional design of this study limits the ability to establish causal relationships between parental styles, EFs difficulties, and anxiety and depression symptoms in adolescents. Although the current findings suggest potential pathways associating these variables, it is important to note that parental style is a dynamic construct that evolves over time, whereas EFs and anxiety and depression symptoms were assessed based on adolescents’ current functioning state at baseline. Therefore, a longitudinal research design would be better suited to capture how changes in parental styles influence the development of EFs and anxiety and depression symptoms over time and how other relevant factors not included in the present study are also likely to contribute to adolescents’ symptomatology. Lastly, participants were nested within five schools, meaning that observations from students attending the same school may not have been fully independent. Although multilevel modeling or cluster‐robust standard errors would ordinarily be considered, previous research indicates that estimates can be unstable or biased when the number of higher level units is very small [106–109]. The school complexity level was therefore included as a contextual covariate, although this does not fully account for within‐school dependence. Finally, future studies should use prospectively determined sample sizes based on formal power analyses for parallel indirect‐effect models and should recruit larger, more balanced school‐vulnerability groups.

It should be noted that adolescence is a period during which significant developmental changes occur in the prefrontal cortex, which are associated with EFs [110]. Many authors have found that these neuroanatomical changes are associated with an increased susceptibility to anxiety and depression symptoms at this age [111], but this age‐related phenomenon might not be as pronounced at other stages of adolescence. Therefore, future studies could complement our model by comparing different age groups across adolescence (early, middle, and late adolescents), comparing clinical and nonclinical populations, and also including the factors that mediate the onset of anxiety and depression symptoms in adolescents and how enhancing EFs could benefit them in a preventive capacity.

In conclusion, this study demonstrates that the impact of social vulnerability and parental styles on adolescent mental health is not direct but is mediated by specific cognitive mechanisms, particularly cognitive flexibility. This finding suggests that positive parental communication may serve as a secure environment that not only fosters EFs but also promotes mentalization or reflective functioning, enabling adolescents to better regulate their internal states. From a public health perspective, these results highlight the need for school‐based interventions that move beyond traditional emotional support by integrating EFs training as a tool for equity to protect the well‐being of adolescents in high‐vulnerability settings.

## Funding

This work was supported by the Agency for the Management of University and Research Grants [AGAUR] under Grant 2023 IMPAC (Grant 00019), by a NARSAD Young Investigator Grant from the Brain & Behavior Research Foundation (Grant 31607), by the MCIN/Agencia Española de Investigación, and by the European Union NextGenerationEU/PRTR (Grant JDC2022‐048939‐I).

## Disclosure

The funders had no role in the design of the study, in the collection, analyses, or interpretation of data, in the writing of the manuscript, or in the decision to publish the results.

## Ethics Statement

The study was conducted in accordance with the Declaration of Helsinki and approved by the Institutional Review Board (or Ethics Committee) of the Universitat Internacional de Catalunya (PSI‐2023‐04; November 15, 2023).

## Consent

Informed consent was obtained from all individual participants included in the study and their legal guardians.

## Conflicts of Interest

The authors declare no conflicts of interest.

## Data Availability

The raw data supporting the conclusions of this article will be made available by the authors upon request.

## References

[1] Diamond A. , Executive Functions, Annual Review of Psychology. (2013) 64, no. 1, 135–168, 10.1146/annurev-psych-113011-143750. PMC4084861 23020641

[2] Funahashi S. , Neuronal Mechanisms of Executive Control by the Prefrontal Cortex, Neuroscience Research. (2001) 39, no. 2, 147–165, 10.1016/S0168-0102(00)00224-8. 11223461

[3] Hughes C. , Graham A. , and Grayson A. , Executive Function in Childhood: Development and Disorder, Cognitive and Language Development in Children, 2004, Open University Press/Blackwell, 205–230.

[4] Miyake A. , Friedman N. P. , Emerson M. J. , Witzki A. H. , Howerter A. , and Wager T. D. , The Unity and Diversity of Executive Functions and Their Contributions to Complex “Frontal Lobe” Tasks: A Latent Variable Analysis, Cognitive Psychology. (2000) 41, no. 1, 49–100, 10.1006/cogp.1999.0734. 10945922

[5] Blain-Brière B. , Bouchard C. , and Bigras N. , The Role of Executive Functions in the Pragmatic Skills of Children Age 4–5, Frontiers in Psychology. (2014) 5, 10.3389/fpsyg.2014.00240, 240. 24688480 PMC3960491

[6] Nouwens S. , Groen M. A. , and Verhoeven L. , How Storage and Executive Functions Contribute to Children’s Reading Comprehension, Learning and Individual Differences. (2016) 47, 96–102, 10.1016/j.lindif.2015.12.008.

[7] Slot P. L. and von Suchodoletz A. , Bidirectionality in Preschool Children’s Executive Functions and Language Skills: Is One Developing Skill the Better Predictor of the Other?, Early Childhood Research Quarterly. (2018) 42, 205–214, 10.1016/j.ecresq.2017.10.005.

[8] Flynn E. , The Role of Inhibitory Control in False Belief Understanding, Infant and Child Development. (2007) 16, no. 1, 53–69, 10.1002/icd.500.

[9] Sabbagh M. A. , Xu F. , Carlson S. M. , Moses L. J. , and Lee K. , The Development of Executive Functioning and Theory of Mind: A Comparison of Chinese and US Preschoolers, Psychological Science. (2006) 17, no. 1, 74–81, 10.1111/j.1467-9280.2005.01667.x. 16371147 PMC2567057

[10] Wang Y. and Liu Y. , The Development of Internalizing and Externalizing Problems in Primary School: Contributions of Executive Function and Social Competence, Child Development. (2021) 92, no. 3, 889–903, 10.1111/cdev.13462. 32857446

[11] Blair C. and Raver C. C. , School Readiness and Self-Regulation: A Developmental Psychobiological Approach, Annual Review of Psychology. (2015) 66, no. 1, 711–731, 10.1146/annurev-psych-010814-015221. PMC4682347 25148852

[12] Cortés Pascual A. , Moyano Muñoz N. , and Quílez Robres A. , The Relationship Between Executive Functions and Academic Performance in Primary Education: Review and Meta-Analysis, Frontiers in Psychology. (2019) 10, 10.3389/fpsyg.2019.01582, 1582. 31354585 PMC6638196

[13] Jacob R. and Parkinson J. , The Potential for School-Based Interventions That Target Executive Function to Improve Academic Achievement: A Review, Review of Educational Research. (2015) 85, no. 4, 512–552, 10.3102/0034654314561338.

[14] Marek S. and Dosenbach N. U. F. , The Frontoparietal Network: Function, Electrophysiology, and Importance of Individual Precision Mapping, Dialogues in Clinical Neuroscience. (2018) 20, no. 2, 133–140, 10.31887/DCNS.2018.20.2/smarek. 30250390 PMC6136121

[15] Benes F. M. , The Development of Prefrontal Cortex: The Maturation of Neurotransmitter Systems and Their Interactions, Handbook of Developmental Cognitive Neuroscience, 2001, MIT Press, 79–92.

[16] Scheibel R. S. and Levin H. S. , Frontal Lobe Dysfunction Following Closed Head Injury in Children: Findings From Neuropsychology and Brain Imaging, Development of Prefrontal Cortex: Evolution, Neurobiology, and Behavior, 1997, Paul H. Brookes Publishing Co., 241–263.

[17] Altenburger L. E. and Schoppe-Sullivan S. J. , Contributions of Parenting Quality and Coparenting Relationship Quality to the Development of Child Executive Functioning, Early Childhood Research Quarterly. (2021) 57, 133–143, 10.1016/j.ecresq.2021.05.010. 36313214 PMC9615140

[18] Bernier A. , Carlson S. M. , and Whipple N. , From External Regulation to Self-Regulation: Early Parenting Precursors of Young Children’s Executive Functioning, Child Development. (2010) 81, no. 1, 326–339, 10.1111/j.1467-8624.2009.01397.x. 20331670

[19] Moriguchi Y. , The Early Development of Executive Function and Its Relation to Social Interaction: A Brief Review, Frontiers in Psychology. (2014) 5, 10.3389/fpsyg.2014.00388, 388. 24808885 PMC4010730

[20] Zelazo P. D. , Blair C. B. , and Willoughby M. T. , Executive Function: Implications for Education, 2016, National Center for Education Research, NCER 2017-2000.

[21] Spruijt A. M. , Dekker M. C. , Ziermans T. B. , and Swaab H. , Educating Parents to Improve Parent–Child Interactions: Fostering the Development of Attentional Control and Executive Functioning, British Journal of Educational Psychology. (2020) 90, no. S1, 158–175, 10.1111/bjep.12312. 31392719 PMC7380015

[22] Koşkulu-Sancar S. , van de Weijer-Bergsma E. , Mulder H. , and Blom E. , Examining the Role of Parents and Teachers in Executive Function Development in Early and Middle Childhood: A Systematic Review, Developmental Review. (2023) 67, 10.1016/j.dr.2022.101063, 101063.

[23] Besharat M. A. , Azizi K. , and Poursharifi H. , The Relationship Between Parenting Styles and Children’s Academic Achievement in a Sample of Iranian Families, Procedia-Social & Behavioral Sciences. (2011) 15, 1280–1283, 10.1016/j.sbspro.2011.03.277.

[24] Coleman P. K. and Karraker K. H. , Self-Efficacy and Parenting Quality: Findings and Future Applications, Developmental Review. (1998) 18, no. 1, 47–85, 10.1006/drev.1997.0448.

[25] Fay-Stammbach T. , Hawes D. J. , and Meredith P. , Parenting Influences on Executive Function in Early Childhood: A Review, Child Development Perspectives. (2014) 8, no. 4, 258–264, 10.1111/cdep.12095.

[26] Sosic-Vasic Z. , Kröner J. , Schneider S. , Vasic N. , Spitzer M. , and Streb J. , The Association Between Parenting Behavior and Executive Functioning in Children and Young Adolescents, Frontiers in Psychology. (2017) 8, 10.3389/fpsyg.2017.00472, 472. 28424644 PMC5371664

[27] Valcan D. S. , Davis H. , and Pino-Pasternak D. , Parental Behaviours Predicting Early Childhood Executive Functions: A Meta-Analysis, Educational Psychology Review. (2018) 30, no. 3, 607–649, 10.1007/s10648-017-9411-9.

[28] Pinquart M. , Associations of Parenting Dimensions and Styles With Internalizing Symptoms in Children and Adolescents: A Meta-Analysis, Marriage & Family Review. (2016) 53, no. 7, 613–640, 10.1080/01494929.2016.1247761.

[29] Pinquart M. , Associations of Parenting Dimensions and Styles With Externalizing Problems of Children and Adolescents: An Updated Meta-Analysis, Developmental Psychology. (2017) 53, no. 5, 873–932, 10.1037/dev0000295. 28459276

[30] Alegre A. , Benson M. J. , and Pérez-Escoda N. , Maternal Warmth and Early Adolescents’ Internalizing Symptoms and Externalizing Behavior: Mediation via Emotional Insecurity, The Journal of Early Adolescence. (2014) 34, no. 6, 712–735, 10.1177/0272431613501408.

[31] Yap M. B. H. , Fowler M. , Reavley N. , and Jorm A. F. , Parenting Strategies for Reducing the Risk of Childhood Depression and Anxiety Disorders: A Delphi Consensus Study, Journal of Affective Disorders. (2015) 183, 330–338, 10.1016/j.jad.2015.05.031. 26047961

[32] Repetti R. L. , Taylor S. E. , and Seeman T. E. , Risky Families: Family Social Environments and the Mental and Physical Health of Offspring, Psychological Bulletin. (2002) 128, no. 2, 330–366, 10.1037/0033-2909.128.2.330. 11931522

[33] Barber B. K. and Harmon E. L. , Violating the Self: Parental Psychological Control of Children and Adolescents, 2002.

[34] Yaffe Y. , A Narrative Review of the Relationship Between Parenting and Anxiety Disorders in Children and Adolescents, International Journal of Adolescence and Youth. (2021) 26, no. 1, 449–459, 10.1080/02673843.2021.1980067.

[35] Liu S. , Hu S. X. , and Su L. , Parental Democratic Communication and Adolescent Well-Being in an Era of Loneliness: The Mediating Role of Societal Trust, Frontiers in Psychiatry. (2024) 15, 10.3389/fpsyt.2024.1500937, 1500937. 39670151 PMC11635610

[36] Estlein R. , Parenting as a Communication Process: Integrating Interpersonal Communication Theory and Parenting Styles Conceptualization, Journal of Family Theory & Review. (2021) 13, no. 1, 21–33, 10.1111/jftr.12407.

[37] Sanavi F. S. , Baghbanian A. , Shovey M. F. , and Ansari-Moghaddam A. , A Study on Family Communication Pattern and Parenting Styles With Quality of Life in Adolescent, Journal of the Pakistan Medical Association. (2013) 63, no. 11, 1393–1398. 24392526

[38] Granero R. , Louwaars L. , and Ezpeleta L. , Socioeconomic Status and Oppositional Defiant Disorder in Preschoolers: Parenting Practices and Executive Functioning as Mediating Variables, Frontiers in Psychology. (2015) 6, 10.3389/fpsyg.2015.01412, 1412. 26441784 PMC4585310

[39] Jiang Y. , Wishard Guerra A. , and Cohen S. R. , et al.Echoing Parental Scaffolding Style in Co–Constructed Narratives: Its Impact on Executive Function Development in Diverse Early School-Age Children, Early Education and Development. (2024) 35, no. 6, 1335–1352.

[40] Khurana A. , Leonard H. , Michaelson L. , and Kosty D. , Transactional Linkages Between Parenting Behaviors and Child Executive Functions and Self-Regulation From Early Childhood to Adolescence, Journal for Person-Oriented Research. (2024) 10, no. 1, 26–55, 10.17505/jpor.2024.26261. 38841560 PMC11149413

[41] Lam C. B. , Chung K. K. H. , and Li X. , Parental Warmth and Hostility and Child Executive Function Problems: A Longitudinal Study of Chinese Families, Frontiers in Psychology. (2018) 9, 10.3389/fpsyg.2018.01063, 1063. 30022960 PMC6040216

[42] Lund J. I. , Toombs E. , Radford A. , Boles K. , and Mushquash C. , Adverse Childhood Experiences and Executive Function Difficulties in Children: A Systematic Review, Child Abuse & Neglect. (2020) 106, 10.1016/j.chiabu.2020.104485, 104485. 32388225

[43] Wade M. , Zeanah C. H. , Fox N. A. , and Nelson C. A. , Global Deficits in Executive Functioning are Transdiagnostic Mediators Between Severe Childhood Neglect and Psychopathology in Adolescence, Psychological Medicine. (2020) 50, no. 10, 1687–1694, 10.1017/S0033291719001764. 31391139 PMC8026012

[44] Lawson G. M. , Duda J. T. , Avants B. B. , Wu J. , and Farah M. J. , Associations Between Children’s Socioeconomic Status and Prefrontal Cortical Thickness, Developmental Science. (2013) 16, no. 5, 641–652, 10.1111/desc.12096. 24033570 PMC3775298

[45] Miller J. G. , López V. , Buthmann J. L. , Garcia J. M. , and Gotlib I. H. , A Social Gradient of Cortical Thickness in Adolescence: Relationships With Neighborhood Socioeconomic Disadvantage, Family Socioeconomic Status, and Depressive Symptoms, Biological Psychiatry Global Open Science. (2022) 2, no. 3, 253–262, 10.1016/j.bpsgos.2022.03.005. 36032055 PMC9410503

[46] Hackman D. A. , Gallop R. , Evans G. W. , and Farah M. J. , Socioeconomic Status and Executive Function: Developmental Trajectories and Mediation, Developmental Science. (2015) 18, no. 5, 686–702, 10.1111/desc.12246. 25659838

[47] Ursache A. , Noble K. G. , and Pediatric Imaging, Neurocognition and Genetics Study , Socioeconomic Status, White Matter, and Executive Function in Children, Brain and Behavior. (2016) 6, no. 10, 10.1002/brb3.531, e00531. 27781144 PMC5064342

[48] Consell Superior d’Avaluació del Sistema Educatiu , Com Classificar Els Centres Educatius Segons LA Complexitat? Departament d’Educació, Generalitat De Catalunya, 2021, https://repositori.educacio.gencat.cat/bitstream/handle/20.500.12694/1353/classificar_centres_educatius_segons_complexitat_2021.pdf.

[49] Schaefer E. S. , A Configurational Analysis of Children’s Reports of Parental Behavior, Journal of Consulting Psychology. (1965) 29, no. 6, 552–557, 10.1037/h0022702. 5846126

[50] Samper P. , Cortés M. T. , Escrivá V. M. , Nácher M. J. , and Tur A. M. , Adaptation of the Child s Report of Parent Behavior Inventory to the Spanish Population, Psicothema. (2006) 18, no. 2, 263–271. 17296042

[51] Valiente R. M. , Magaz A. , Chorot P. , and Sandín B. , Factor Structure of the Parenting Styles Perception Questionnaire CRPBI-Abbreviated, Journal of Clinical Psychology With Children and Adolescents. (2016) 3, no. 2, 69–78.

[52] Maldonado M. J. , Fournier C. , Martínez R. , González J. , Espejo-Saavedra J. M. , and Santamaría P. , BRIEF-2. Evaluación Conductual de La Función Ejecutiva [BRIEF-2. Behavioral Assessment of the Executive Functioning], 2017, TEA Ediciones.

[53] Chorpita B. F. , Yim L. , Moffitt C. , Umemoto L. A. , and Francis S. E. , Assessment of Symptoms of DSM-IV Anxiety and Depression in Children: A Revised Child Anxiety and Depression Scale, Behaviour Research and Therapy. (2000) 38, no. 8, 835–855, 10.1016/S0005-7967(99)00130-8. 10937431

[54] Sandín B. , Valiente R. M. , and Chorot P. , RCADS: Assessment of Symptoms of Anxiety and Depressive Disorders in Children and Adolescents, Journal of Psychopathology and Clinical Psychology. (2009) 14, no. 3, 193–206.

[55] Sandín B. , Chorot P. , Valiente R. M , and Chorpita B. F. , Desarrollo de una versión de 30 ítems de la Revised Child Anxiety and Depression Scale, Revista de Psicopatología y Psicología Clínica. (2010) 15, no. 3, 165–178, 10.5944/rppc.vol.15.num.3.2010.4095.

[56] Lemstra M. , Neudorf C. , D’Arcy C. , Kunst A. , Warren L. M. , and Bennett N. R. , A Systematic Review of Depressed Mood and Anxiety by SES in Youth Aged 10–15 Years, Canadian Journal of Public Health. (2008) 99, no. 2, 125–129, 10.1007/BF03405459. 18457287 PMC6975760

[57] Chen E. and Miller G. E. , Socioeconomic Status and Health: Mediating and Moderating Factors, Annual Review of Clinical Psychology. (2013) 9, no. 1, 723–749, 10.1146/annurev-clinpsy-050212-185634. 23245339

[58] Rowe M. L. , Coker D. , and Pan B. A. , A Comparison of Fathers’ and Mothers’ Talk to Toddlers in Low-Income Families, Social Development. (2004) 13, no. 2, 278–291, 10.1111/j.1467-9507.2004.000267.x.

[59] Hackman D. A. and Farah M. J. , Socioeconomic Status and the Developing Brain, Trends in Cognitive Sciences. (2009) 13, no. 2, 65–73, 10.1016/j.tics.2008.11.003. 19135405 PMC3575682

[60] Hoffman L. W. , Methodological Issues in Studies of SES, Parenting, and Child Development, Socioeconomic Status, Parenting, and Child Development, 2014, Routledge, 125–143.

[61] Roubinov D. S. and Boyce W. T. , Parenting and SES: Relative Values or Enduring Principles?, Current Opinion in Psychology. (2017) 15, 162–167, 10.1016/j.copsyc.2017.03.001. 28503655 PMC5423399

[62] Lawson G. M. , Hook C. J. , and Farah M. J. , A Meta-Analysis of the Relationship Between Socioeconomic Status and Executive Function Performance Among Children, Developmental Science. (2018) 21, no. 2, 10.1111/desc.12529, e12529. PMC5821589 28557154

[63] Rakesh D. , Lee P. A. , Gaikwad A. , and McLaughlin K. A. , Annual Research Review: Associations of Socioeconomic Status With Cognitive Function, Language Ability, and Academic Achievement in Youth: A Systematic Review of Mechanisms and Protective Factors, Journal of Child Psychology and Psychiatry. (2025) 66, no. 4, 417–439, 10.1111/jcpp.14082. 39625804 PMC11920614

[64] Fontaine G. P. , Blake K. V. , Koen N. , Stein D. J. , Hammar Å. , and Groenewold N. A. , Executive Functioning in Adolescents With Internalizing Disorders: A Systematic Review, European Child & Adolescent Psychiatry. (2026) 35, no. 2, 341–364, 10.1007/s00787-025-02826-2. 41081899 PMC12957021

[65] Alves M. , Yamamoto T. , and Arias-Carrion O. , et al.Executive Function Impairments in Patients With Depression, CNS & Neurological Disorders - Drug Targets. (2014) 13, no. 6, 1026–1040, 10.2174/1871527313666140612102321. 24923347

[66] Malloy-Diniz L. F. , Miranda D. M. , and Grassi-Oliveira R. , Editorial: Executive Functions in Psychiatric Disorders, Frontiers in Psychology. (2017) 8, 10.3389/fpsyg.2017.01461, 1461. 28928684 PMC5591960

[67] Warren S. L. , Heller W. , and Miller G. A. , The Structure of Executive Dysfunction in Depression and Anxiety, Journal of Affective Disorders. (2021) 279, 208–216, 10.1016/j.jad.2020.09.132. 33059224 PMC7738359

[68] Liu G. , Zhang N. , and Teoh J. Y. , et al.Self-Compassion and Dorsolateral Prefrontal Cortex Activity During Sad Self-Face Recognition in Depressed Adolescents, Psychological Medicine. (2022) 52, no. 5, 864–873, 10.1017/S0033291720002482. 32698918 PMC8208230

[69] Walters M. and Hines-Martin V. , Overview of Executive Functions in Mood and Depressive Disorders: A Review of the Literature, Archives of Psychiatric Nursing. (2018) 32, no. 4, 617–637, 10.1016/j.apnu.2018.02.011. 30029757

[70] Qiu Z. , Guo Y. , Wang J. , and Zhang H. , Associations of Parenting Style and Resilience With Depression and Anxiety Symptoms in Chinese Middle School Students, Frontiers in Psychology. (2022) 13, 10.3389/fpsyg.2022.897339, 897339. 35846635 PMC9285101

[71] Keijser R. , Olofsdotter S. , Nilsson K. W. , and Åslund C. , The Influence of Parenting Styles and Parental Depression on Adolescent Depressive Symptoms: A Cross-Sectional and Longitudinal Approach, Mental Health & Prevention. (2020) 20, 10.1016/j.mhp.2020.200193, 200193.

[72] Graziano P. A. , Calkins S. D. , and Keane S. P. , Sustained Attention Development During the Toddlerhood to Preschool Period: Associations With Toddlers’ Emotion Regulation Strategies and Maternal Behavior, Infant and Child Development. (2011) 20, no. 6, 389–408, 10.1002/icd.731. 22121338 PMC3222561

[73] Schäfer J.Ö. , Naumann E. , Holmes E. A. , Tuschen-Caffier B. , and Samson A. C. , Emotion Regulation Strategies in Depressive and Anxiety Symptoms in Youth: A Meta-Analytic Review, Journal of Youth and Adolescence. (2017) 46, no. 2, 261–276, 10.1007/s10964-016-0585-0. 27734198

[74] Katsantonis I. and Symonds J. E. , Population Heterogeneity in Developmental Trajectories of Internalising and Externalising Mental Health Symptoms in Childhood: Differential Effects of Parenting Styles, Epidemiology and Psychiatric Sciences. (2023) 32, 10.1017/S2045796023000094, e16. 36999252 PMC10130732

[75] Pinquart M. and Gerke D.-C. , Associations of Parenting Styles With Self-Esteem in Children and Adolescents: A Meta-Analysis, Journal of Child and Family Studies. (2019) 28, no. 8, 2017–2035, 10.1007/s10826-019-01417-5.

[76] Liang L. , Zhang N. , Liu W. , Lin L. , and Zhang X. , Longitudinal Relations Between Maternal Parenting Styles and Preschoolers’ Externalizing Problem Behaviors: A Chain Mediation Model, Child & Youth Care Forum. (2025) 54, no. 2, 453–470.

[77] Herd T. , Brieant A. , King-Casas B. , and Kim-Spoon J. , Associations Between Developmental Patterns of Negative Parenting and Emotion Regulation Development Across Adolescence, Emotion. (2022) 22, no. 2, 270–282, 10.1037/emo0000997. 34435842 PMC8881298

[78] Toth S. L. , Cicchetti D. , Macfie J. , and Emde R. N. , Representations of Self and Other in the Narratives of Neglected, Physically Abused, and Sexually Abused Preschoolers, Development and Psychopathology. (1997) 9, no. 4, 781–796, 10.1017/S0954579497001430. 9449005

[79] Hildyard K. L. and Wolfe D. A. , Child Neglect: Developmental Issues and Outcomes☆, Child Abuse & Neglect. (2002) 26, no. 6-7, 679–695, 10.1016/S0145-2134(02)00341-1. 12201162

[80] Williams K. E. , Ciarrochi J. , and Heaven P. C. L. , Inflexible Parents, Inflexible Kids: A 6-Year Longitudinal Study of Parenting Style and the Development of Psychological Flexibility in Adolescents, Journal of Youth and Adolescence. (2012) 41, no. 8, 1053–1066, 10.1007/s10964-012-9744-0. 22311519

[81] Roberts H. , Schreiner M. W. , and Pocius S. , et al.State Rumination Predicts Inhibitory Control Failures and Dysregulation of Default, Salience, and Cognitive Control Networks in Youth at Risk of Depressive Relapse: Findings From the RuMeChange Trial, Journal of Affective Disorders Reports. (2024) 16, 10.1016/j.jadr.2024.100729, 100729. 38769946 PMC11105748

[82] Wagner C. A. , Alloy L. B. , and Abramson L. Y. , Trait Rumination, Depression, and Executive Functions in Early Adolescence, Journal of Youth and Adolescence. (2015) 44, no. 1, 18–36, 10.1007/s10964-014-0133-8. 24839132 PMC4236277

[83] Shimony O. , Einav N. , Bonne O. , Jordan J. T. , Van Vleet T. M. , and Nahum M. , The Association Between Implicit and Explicit Affective Inhibitory Control, Rumination and Depressive Symptoms, Scientific Reports. (2021) 11, no. 1, 10.1038/s41598-021-90875-3, 11490. 34075112 PMC8169859

[84] Rose E. J. and Ebmeier K. P. , Pattern of Impaired Working Memory During Major Depression, Journal of Affective Disorders. (2006) 90, no. 2-3, 149–161, 10.1016/j.jad.2005.11.003. 16364451

[85] Hitchcock C. , Hammond E. , and Rees C. , et al.Memory Flexibility Training (MemFlex) to Reduce Depressive Symptomatology in Individuals With Major Depressive Disorder: Study Protocol for a Randomised Controlled Trial, Trials. (2015) 16, no. 1, 1–8, 10.1186/s13063-015-1029-y. 26531124 PMC4632349

[86] Vedechkina M. , Bennett M. , and Holmes J. , Dimensions of Internalizing Symptoms are Stable Across Early Adolescence and Predicted by Executive Functions: Longitudinal Findings From the Adolescent Brain and Cognitive Development (ABCD) Study, Development and Psychopathology. (2024) 36, no. 3, 1284–1293, 10.1017/S0954579423000524. 37272416

[87] Stange J. P. , Alloy L. B. , and Fresco D. M. , Inflexibility as a Vulnerability to Depression: A Systematic Qualitative Review, Clinical Psychology: Science and Practice. (2017) 24, no. 3, 245–276, 10.1111/cpsp.12201. 29038622 PMC5640320

[88] Haag A.-C. , Bagrodia R. , and Bonanno G. A. , Emotion Regulation Flexibility in Adolescents: A Systematic Review From Conceptualization to Methodology, Clinical Child and Family Psychology Review. (2024) 27, no. 3, 697–713, 10.1007/s10567-024-00483-6. 39003663 PMC11486788

[89] Fombouchet Y. , Lannegrand L. , and Lucenet J. , Relationships Between Emotion Regulation Strategies and Executive Functions in Adolescence: Exploring the Effects of Discrete Emotions and Age, Journal of Adolescence. (2024) 96, no. 6, 1239–1248, 10.1002/jad.12334. 38693714

[90] Ratliff E. L. , Morris A. S. , Cui L. , Jespersen J. E. , Silk J. S. , and Criss M. M. , Supportive Parent-Adolescent Relationships as a Foundation for Adolescent Emotion Regulation and Adjustment, Frontiers in Psychology. (2023) 14, 10.3389/fpsyg.2023.1193449, 1193449. 37546468 PMC10400008

[91] Cardoso A. S. , Martins R. C. , Venturin B. , Gonzalez A. , Blumenberg C. , and Murray J. , Psychosocial Adversity and Executive Functions in Children and Adolescents: A Systematic Review of Mediating and Moderating Influences of Parenting Behaviors, BMC Psychiatry. (2025) 25, no. 1, 10.1186/s12888-025-06592-y, 894. 41029261 PMC12487171

[92] Sharp C. and Fonagy P. , The Parent’s Capacity to Treat the Child as a Psychological Agent: Constructs, Measures and Implications for Developmental Psychopathology, Social Development. (2008) 17, no. 3, 737–754, 10.1111/j.1467-9507.2007.00457.x.

[93] Vučković S. , Ručević S. , and Ajduković M. , Parenting Style and Practices and Children’s Externalizing Behavior Problems: Mediating Role of Children’s Executive Functions, European Journal of Developmental Psychology. (2021) 18, no. 3, 313–329, 10.1080/17405629.2020.1768067.

[94] Fatima S. , Sheikh H. , and Ardila A. , Association of Parent-Child Relationships and Executive Functioning in South Asian Adolescents, Neuropsychology. (2016) 30, no. 1, 65–74, 10.1037/neu0000216. 26052717

[95] Diamond A. and Lee K. , Interventions Shown to Aid Executive Function Development in Children 4 to 12 Years Old, Science. (2011) 333, no. 6045, 959–964, 10.1126/science.1204529. 21852486 PMC3159917

[96] Hofmann W. , Schmeichel B. J. , and Baddeley A. D. , Executive Functions and Self-Regulation, Trends in Cognitive Sciences. (2012) 16, no. 3, 174–180, 10.1016/j.tics.2012.01.006. 22336729

[97] Zelazo P. D. and Cunningham W. A. , Executive Function: Mechanisms Underlying Emotion Regulation, 2007.

[98] Young K. , Sandman C. , and Craske M. , Positive and Negative Emotion Regulation in Adolescence: Links to Anxiety and Depression, Brain Sciences. (2019) 9, no. 4, 10.3390/brainsci9040076, 76. 30934877 PMC6523365

[99] Kraft L. , Ebner C. , Leo K. , and Lindenberg K. , Emotion Regulation Strategies and Symptoms of Depression, Anxiety, Aggression, and Addiction in Children and Adolescents: A Meta-Analysis and Systematic Review, Clinical Psychology: Science and Practice. (2023) 30, no. 4, 485–502.

[100] Diamond A. , Activities and Programs that Improve Children’s Executive Functions, Current Directions in Psychological Science. (2012) 21, no. 5, 335–341, 10.1177/0963721412453722. 25328287 PMC4200392

[101] Diamond A. and Ling D. S. , Conclusions About Interventions, Programs, and Approaches for Improving Executive Functions That Appear Justified and Those That, Despite Much Hype, Do Not, Developmental Cognitive Neuroscience. (2016) 18, 34–48, 10.1016/j.dcn.2015.11.005. 26749076 PMC5108631

[102] Oakes J. M. and Rossi P. H. , The Measurement of SES in Health Research: Current Practice and Steps Toward a New Approach, Social Science & Medicine. (2003) 56, no. 4, 769–784, 10.1016/S0277-9536(02)00073-4. 12560010

[103] Pino Muñoz M. and Arán Filippetti V. , Confirmatory Factor Analysis of the BRIEF-2 Parent and Teacher Form: Relationship to Performance-Based Measures of Executive Functions and Academic Achievement, Applied Neuropsychology: Child. (2021) 10, no. 3, 219–233, 10.1080/21622965.2019.1660984. 31522525

[104] Kröner-Herwig B. , Morris L. , Heinrich M. , Gassmann J. , and Vath N. , Agreement of Parents and Children on Characteristics of Pediatric Headache, Other Pains, Somatic Symptoms, and Depressive Symptoms in an Epidemiologic Study, The Clinical Journal of Pain. (2009) 25, no. 1, 58–64, 10.1097/AJP.0b013e31817fc62d. 19158547

[105] Leve L. D. , DeGarmo D. S. , and Bridgett D. J. , et al.Using an Adoption Design to Separate Genetic, Prenatal, and Temperament Influences on Toddler Executive Function, Developmental Psychology. (2013) 49, no. 6, 1045–1057, 10.1037/a0029390. 22799580 PMC3509265

[106] Colin Cameron A. and Miller D. L. , A Practitioner’s Guide to Cluster-Robust Inference, Journal of Human Resources. (2015) 50, no. 2, 317–372, 10.3368/jhr.50.2.317.

[107] Huang F. L. and Li X. , Using Cluster-Robust Standard Errors When Analyzing Group-Randomized Trials With Few Clusters, Behavior Research Methods. (2022) 54, no. 3, 1181–1199, 10.3758/s13428-021-01627-0. 34505994

[108] McNeish D. M. and Stapleton L. M. , The Effect of Small Sample Size on Two-Level Model Estimates: A Review and Illustration, Educational Psychology Review. (2016) 28, no. 2, 295–314, 10.1007/s10648-014-9287-x.

[109] McNeish D. and Stapleton L. M. , Modeling Clustered Data With Very Few Clusters, Multivariate Behavioral Research. (2016) 51, no. 4, 495–518, 10.1080/00273171.2016.1167008. 27269278

[110] Raznahan A. , Shaw P. , and Lalonde F. , et al.How Does Your Cortex Grow?, The Journal of Neuroscience. (2011) 31, no. 19, 7174–7177, 10.1523/JNEUROSCI.0054-11.2011. 21562281 PMC3157294

[111] Paus T. , Keshavan M. , and Giedd J. N. , Why Do Many Psychiatric Disorders Emerge During Adolescence?, Nature Reviews Neuroscience. (2008) 9, no. 12, 947–957, 10.1038/nrn2513. 19002191 PMC2762785

